# Generalisability of Maternal Genetic Risk Score for Birth Weight Across Racial Identity and Ancestry: A Secondary Analysis of a Prospective Cohort Study

**DOI:** 10.1111/1471-0528.70088

**Published:** 2025-11-30

**Authors:** Bita Tristani‐Firouzi, Lisa Pappas, Merry Joseph, Maryam Zeinomar, Michelle P. Debbink, Joseph Mims, Rafael Guerrero, Barry Moore, Robert M. Silver, Tsegaselassie Workalemahu, David Haas, Jonathan G. Steller, George Saade, Nathan R. Blue

**Affiliations:** ^1^ Department of Obstetrics and Gynecology University of Utah Health Salt Lake City Utah USA; ^2^ Intermountain Health Salt Lake City Utah USA; ^3^ Department of Biological Sciences North Carolina State University Raleigh North Carolina USA; ^4^ Department of Human Genetics University of Utah Salt Lake City Utah USA; ^5^ Department of Obstetrics and Gynecology Indiana University Indianapolis Indiana USA; ^6^ Department of Obstetrics and Gynecology, Division of Maternal Fetal Medicine University of California, Irvine Orange California USA; ^7^ Department of Obstetrics and Gynecology Eastern Virginia Medical School Norfolk Virginia USA

**Keywords:** equity, foetal growth, genetic risk score, polygenic risk score

## Abstract

**Objective:**

Maternal genotypes may be useful to customise foetal growth assessment, but generalisability across diverse racial and ancestral groups remains uncertain. We assessed the generalisability of a genetic risk score for birth weight (GRS_BW_), derived from participants of predominantly European ancestry, within a diverse U.S. cohort.

**Design:**

Secondary analysis of a prospective observational cohort of nulliparous patients.

**Setting:**

Eight U.S. recruitment centers.

**Population or Sample:**

Participants in the parent study with available maternal DNA.

**Methods:**

We used log‐linear modelling to test the association of maternal GRS_BW_ with infant birth weight. We then assessed the robustness of the association by self‐identified race and genetically predicted continental ancestry (GPA) groups.

**Main Outcome Measures:**

Birth weight.

**Results:**

Among 8147 eligible participants, GRS_BW_ was positively associated with birth weight (*p* < 0.001). Across self‐identified racial groups, the association was significant in White (*n* = 5394, mean ratio 1.04, 95% CI 0.97–1.11, *p* = 0.007) and multiracial (*n* = 508, mean ratio 1.10, CI 1.01–1.2, *p* = 0.03) groups but not in Black (*n* = 1139), Asian (*n* = 358), or unknown race groups (*p* > 0.09 for all). Among GPA groups, the association was significant among European (mean ratio 1.04, CI 1.02–1.07, *p* = 0.001) and American (mean ratio 1.08, CI 1.01–1.14, *p* = 0.02) ancestry groups but not African, East or South Asian, or unknown ancestry (*p* > 0.05 for all).

**Conclusions:**

This GRS_BW_ is not generalisable across self‐described racial identities or GPA groups, highlighting the need for globally representative genetic discovery cohorts as well as further investigation into the complex role of race, ethnicity, and epigenetic influences on foetal growth.

## 1 Introduction

Foetal growth restriction (FGR), traditionally defined as estimated birth weight < 10th percentile, is a leading risk factor for stillbirth and a major focus of antenatal ultrasound use [1, 2]. However, current diagnostic strategies for FGR poorly predict perinatal morbidity and mortality, such that most foetuses diagnosed with FGR do not experience any perinatal morbidity [3]. Efforts have been made to customise foetal growth assessments using maternal and foetal factors that are associated with variation in foetal growth, including maternal race [4, 5]. However, race is a socially defined construct that categorises individuals, typically on the basis of physical qualities (e.g., skin colour, hair texture). Within societies, racial categorization creates and reinforces power structures that dictate differential access to opportunity and resources. As a means of justifying withholding resources and power, it is often implied that racial categories are based on inherent biological or genetic realities. However, there is greater genetic variation within racial groups than that found across racial groups, underlining the problematic nature of race as a proxy for genetic, or physiologic, growth potential [6]. Furthermore, using maternal race to customise foetal growth curves has the potential to exacerbate disparities in foetal growth outcomes by conflating the detrimental impacts of socio‐structural marginalisation with inherent genetic growth potential. Potentially reclassifying abnormal growth as normal in this way could result in withholding necessary surveillance and interventions [7, 8].

Instead, the integration of parental genetic data may be a more valid and effective approach to personalise foetal growth assessments, improving recognition of abnormal growth and separating it from nonpathological constitutional smallness. Recent studies have identified genetic markers associated with foetal growth that could be used for such purposes [9, 10, 11]. However, these genetic markers have been identified using exclusively European cohorts. Increasingly, genetic studies demonstrate that for many conditions, genetic findings in predominantly European cohorts do not perform as well in more diverse populations [12, 13, 14]. Therefore, the objective of this study was to assess a previously published genetic risk score (GRS) for birth weight (GRS_BW_), recently developed from a European cohort, for generalizability within groups defined by self‐identified race and genetically predicted ancestry [9].

Because the rationale to use a GRS_BW_ to customise foetal growth assessment is to obviate the impulse to customise growth assessments using maternal race/ethnicity, our secondary objective was to determine whether self‐identified race/ethnicity remains associated with birth weight (BW) after accounting for the GRS_BW_.

## 2 Methods

### 2.1 Study Setting and Population

Our study was a secondary analysis of the Nulliparous Pregnancy Outcomes Study: Monitoring Mothers‐to‐Be (nuMoM2b), a large prospective observational cohort study designed to assess contributors to adverse pregnancy outcomes. Detailed nuMoM2b protocols were previously published and are briefly summarised here [15]. Participants in the parent study were recruited at 8 geographically diverse U.S. sites from 2010 to 2013 and were included if they had a singleton pregnancy between 6 weeks 0 days and 13 weeks 6 days' gestation and no prior pregnancies lasting 20 weeks or more. Potential participants were excluded for age < 13 years, 3 or more prior miscarriages, suspected foetal malformation at the time of enrolment, known foetal aneuploidy, conception using a donor oocyte, multi‐foetal reduction, plan for pregnancy termination, or participation in an intervention study to influence pregnancy outcomes. Participants had 4 study visits: during approximately the first, second, and early third trimesters of pregnancy, as well as one after delivery. For this secondary analysis, we included all participants with a live birth at ≥ 24 weeks with available maternal single nucleotide polymorphism (SNP) array data, derived from unselected maternal blood collection that was part of the protocol for all parent study participants. Participants were excluded if they did not complete any of the 3 research ultrasounds or were missing key variables including infant sex and BW.

### 2.2 Outcome Variable

The primary outcome of this study was infant BW. Clinically measured BW values, typically performed within an hour of birth, were abstracted from medical records by centrally trained research personnel.

### 2.3 Independent Variables

Our primary independent variables of interest included race, genetic ancestry, and GRS_BW_. Participants self‐selected their race designation(s) from among the following choices: White, Black/African American, Asian, Native Hawaiian/Other Pacific Islander, American Indian/Alaskan Native, Multiracial, and Unknown/not reported. Genetic ancestry was ascertained using *Peddy*, a software package that uses an individual's DNA to predict the predominant continental ancestry [16] with the following categorical outputs: AFR, African; AMR, American (Indigenous); EAS, East Asian; EUR, European; SAS, South Asian; UNK, unknown. We assessed the distribution of predicted genetic ancestry within self‐identified racial groups.

GRS_BW_ was calculated using maternal DNA isolated from blood collected at visit 1. Genotyping was performed using a commercially available kit (Infinium Multi‐Ethnic Global D2 Bead Chip; Illumina), from which SNP arrays were analysed based on the Genome Reference Consortium human build 38 (CRCh38) [17]. In 2020, Chen et al. used maternal and infant DNA from *N* = 10 734 maternal–infant duos of European ancestry to identify SNPs that are independently associated with birth weight [9]. They identified and published 86 SNPs (GRCh37) and their magnitudes of association. We mapped these 86 SNPs to GRCh38 for compatibility, yielding 72 SNPs, which we used to compute maternal GRS_BW_ as follows [9]. The list of 72 SNPs and their weights is available in Table S1. The weighted sum of BW‐associated SNPs present in each person was computed, such that the score represents the cumulative effect size without traditional units, expressed as GRS = (*V*_1_*β_1_) + (*V*_2_*β_1_) + … (*V*_72_*β_72_), where *V*_1_ is variant 1 and *β*_1_ is the effect size for variant 1. We then used the GRS_BW_ as a term in a regression model for infant birth weight, which is described below.

Other variables in this analysis included gestational age at delivery and infant sex, both abstracted from medical records by centrally trained research personnel.

### 2.4 Statistical Analysis

Because our preliminary analysis showed that BW variability changes across gestational age, we used a log‐linear model to test the association between maternal GRS_BW_ and infant BW, which addresses this heteroscedasticity better than generalised linear models. We included gestational age at birth and infant sex as a priori variables in the model. We included gestational age because BW is highly dependent on gestational duration, and the SNP weights were derived after adjusting for gestational age, making them independent of gestational age [9]. We included infant sex because prior work shows that sex is strongly associated with foetal growth [18]. We also included sex because none of the BW‐associated SNPs were on sex chromosomes and so would not be expected to account for sex‐related differences in growth. To assess the generalizability of the GRS_BW_ across self‐identified racial groups, the association between GRS_BW_ and BW was assessed for each self‐identified racial subgroup using stratified log‐linear models. This same approach was repeated across groups defined by genetic continental ancestry. GRS_BW_ effect sizes are expressed as the exponentiated ꞵ value mean ratios, or the factor increase in BW for each 1‐unit increase in GRS_BW_. Self‐identified race and genetic ancestry were included as predictor variables in separate log‐linear models to test whether they remained independently associated with BW after controlling for GRS_BW_, infant sex, and gestational age. Finally, to determine whether effect modification was present, we separately added GRS_BW_‐race and GRS_BW_‐ancestry interaction terms and compared the model without versus the model with the interaction terms using a likelihood ratio test.

## 3 Results

**FIGURE 1:**
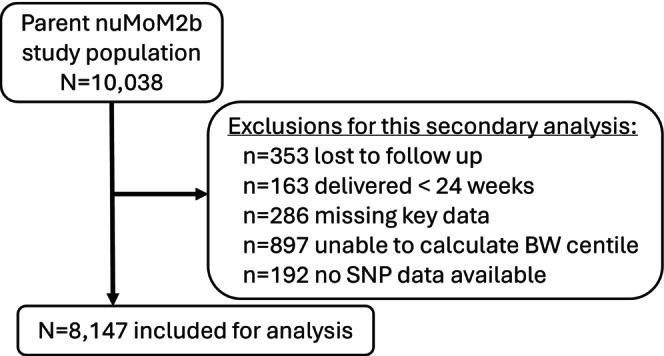
Inclusion flow diagram.

There were 8147 participants that met inclusion criteria (Figure 1). Participants' demographic and obstetric characteristics are shown in Table 1. Table S2 shows background characteristics stratified by self‐identified race. Maternal GRS_BW_ values ranged from −0.214 to 0.713 and were positively associated with infant BW (*p* < 0.0001). However, the magnitude of association (mean ratio) was small at 1.07, meaning that a change in GRS_BW_ of 1.0 (essentially the entire range of possible GRS_BW_ values) is associated with a 7% increase in BW. The log‐linear measures of association for terms in the initial model are shown in Table S3.

**TABLE 1:** Demographic and obstetric characteristics of nuMoM2b participants meeting secondary analysis inclusion criteria.

	*N* = 8147
GA at delivery (weeks), mean (SD)	39.0 (1.92)
Maternal BMI, *n* (%)	
< 18.5 (underweight)	183 (2.2)
18.5 to < 25 (normal weight)	4097 (50.3)
25 to < 30 (overweight)	1964 (24.1)
30 to < 35 (obese)	957 (11.7)
35+ (morbidly obese)	807 (9.9)
Missing	139 (1.7)
Diabetes, *n (%)*	
Pre‐gestational	141 (1.7)
Gestational	372 (4.6)
Chronic hypertension, *n* (%)	225 (2.8)
HDP, *n (%)*	
Gestational HTN	1169 (14.3)
PE without severe features	342 (4.2)
PE with severe features	363 (4.5)
Superimposed PE	47 (0.6)
Eclampsia	5 (0.1)
Birth weight (g), mean (SD)	3239.5 (559.1)
BW < 10th percentile, *n* (%)	773 (9.5)
BW > 90th percentile, *n* (%)	351 (4.3)
Newborn sex male, *n* (%)	4190 (51.4)

Among the included nuMoM2b participants, the largest self‐identified racial group was White (*n* = 5394, 64.1%), followed by Black/African American (*n* = 1139, 14.0%), unknown (*n* = 699, 8.6%), multiracial (*n* = 508, 6.2%), and Asian (*n* = 358, 4.4%). Genetically predicted continental ancestry groups, in order of decreasing size, were EUR (*n* = 5099, 62.6%), AFR (*n* = 1383, 17.0%), AMR (*n* = 1028, 12.6%), EAS (*n* = 274, 3.4%), UNK (*n* = 264, 3.2%), and SAS (*n* = 99, 1.2%). Within each self‐identified racial group, the most common genetic ancestry was as follows: White: EUR (91.6%); Black/African American: AFR (98.8%); Unknown/not reported: AMR (69.4%); multiracial: AFR (30.1%); Asian: EAS (66.5%); Native Hawaiian/Pacific Islander: EAS (68.8%); American Indian/Alaska Native: AMR (76.5%). Overlap between race and predicted ancestry is reported in Table 2.

**TABLE 2:** Alignment between self‐identified race and genetically predicted ancestry in a nulliparous cohort of pregnant people.

All participants, *N* (%)	White	Black/African American	Multi‐racial	Asian	Native Hawaiian/Other Pacific Islander	American Indian/Alaska Native	Unknown
	*n* (%)	*n* (%)	*n* (%)	*n* (%)	*n* (%)	*n* (%)	*n* (%)
*n* = 8147	5394 (66.2)	1139 (14.0)	508 (6.2)	358 (4.4)	32 (0.4)	17 (0.2)	699 (8.6)
Genetic ancestry (*N* %)							
EUR 5099 (62.6)	4949 (91.8)	6 (0.5)	111 (21.9)	1 (0.3)	1 (3.1)	1 (5.9)	30 (4.3)
AFR 1383 (17.0)	8 (0.2)	1125 (98.8)	153 (30.1)	2 (0.6)	2 (6.3)	3 (17.7)	90 (12.9)
AMR 1028 (12.6)	388 (7.2)	4 (0.4)	118 (23.2)	16 (4.5)	4 (12.5)	13 (76.5)	485 (69.4)
EAS 274 (3.4)	1 (0)	0 (0)	9 (1.8)	238 (66.5)	22 (68.8)	0 (0)	4 (0.6)
UNK 264 (3.2)	48 (0.9)	3 (0.3)	114 (22.4)	10 (2.8)	3 (9.4)	0 (0)	86 (12.3)
SAS 99 (1.2)	0 (0)	1 (0.1)	3 (0.6)	91 (25.4)	0 (0)	0 (0)	4 (0.6)

*Note:* Percentages for genetic ancestry within each self‐identified race use the total *n* from the self‐identified race as the denominator. *Peddy* predicts the predominant continental genetic ancestry in single categories. Percentages for total genetic ancestry (far left column) use the overall *N* (8147) as the denominator.

Abbreviations: AFR, African; AMR, American; EAS, East Asian; EUR, European; SAS, South Asian; UNK, Unknown.

**FIGURE 2:**
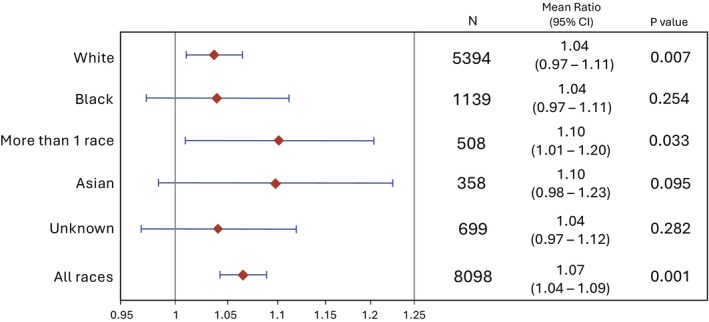
Association of GRS_BW_ with BW in self‐identified radical groups. The forest plot shows the range of the magnitudes of association, with diamonds reflecting the mean ratios of the exponentiated beta coefficients and error bars reflecting the 95% confidence intervals from multi‐variable log‐linear modelling. A mean ratio of 1.04 means that in the model, an increase in GRS_BW_ of 1.0 is associated with an increase in BW of 4% in the White race participants, for example.

Figure 2 shows the results of our primary analysis assessing the generalizability of the association between GRS_BW_ and infant BW by self‐identified race. The association between GRS_BW_ and infant BW was only significant among participants who self‐identified as White (mean ratio 1.04, *p* = 0.007) or more than one race (mean ratio 1.10, *p* = 0.03). Here, a mean ratio of 1.04 means that every one‐unit increase in GRS is associated with an increase in BW of 4%, or a factor of 1.04.

Across racial groups, the magnitude of the association between GRS_BW_ and infant weight varied widely; the expected increase in BW for each 1‐unit increase in GRS in Asian and multi‐racial groups (10.0% for both) was more than double that of White and Black groups (4% for both). The variation in the magnitude of the association and statistical association across racial groups is shown in Figure 2.

**FIGURE 3:**
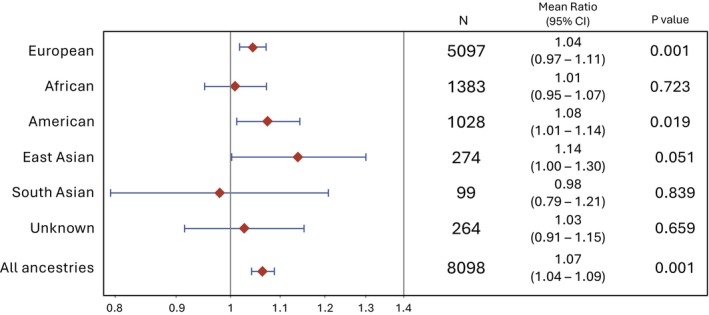
Association of GRS_BW_ with BW in genetically predicted continental ancestry groups. The forest plot shows the range of the magnitudes of association, with diamonds reflecting the mean ratios of the exponentiated beta coefficients and error bars reflecting the 95% confidence intervals from multi‐variable log‐linear modelling. A mean ratio of 1.04 means that in the model, an increase in GRS_BW_ of 1.0 is associated with an increase in BW of 4% in the EUR group, for example.

Figure 3 demonstrates the results for analyses assessing the association between GRS_BW_ and BW within genetically predicted ancestry groups rather than self‐identified race. GRS_BW_ was associated with BW in the EUR (mean ratio 1.04, *p* = 0.001) and AMR (mean ratio 1.08, *p* = 0.02) ancestry groups but not AFR, EAS, SAS, or unknown groups (Figure 3). We did not find any evidence of GRS_BW_‐race or GRS_BW_‐ancestry effect modification when comparing the initial models (in Figures 2 and 3) with models with GRS_BW_‐race or GRS_BW_‐ancestry interaction terms (likelihood ratio tests *p* = 0.8 and 0.5, respectively).

Two final log‐linear models each assessed the association between self‐identified race and genetically predicted ancestry with BW after controlling for GRS_BW_ in the entire included cohort, using the largest groups in each category (White race and EUR predicted ancestry, respectively) as the referent groups. For all groups except American Indian/Alaska Native, self‐identified race was independently associated with lower BW after controlling for GRS_BW_, gestational age, and infant sex (Table 3). All genetically predicted ancestry groups except for UNK remained independently associated with lower BW (Table 3).

**TABLE 3:** Mean ratio estimates for associations between self‐identified race or genetically predicted ancestry with BW after controlling for GRS_BW_.

	Estimate (95% CI)	*p*
Self‐identified race (ref: White)		
American Indian/Alaskan Native	0.99 (0.93–1.05)	0.76
Asian	0.96 (0.95–0.98)	< 0.0001
Black/African American	0.96 (0.95–0.97)	< 0.0001
More than one race	0.99 (0.97–1.0)	0.011
Native Hawaiian/Pacific Islander	0.95 (0.91–1.0)	0.023
Unknown	0.98 (0.97–0.99)	< 0.0001
Genetically predicted continental ancestry (ref: EUR)		
AFR	0.96 (0.95–0.97)	< 0.0001
AMR	0.98 (0.97–0.99)	< 0.0001
EAS	0.98 (0.97–1.0)	0.012
SAS	0.91 (0.89–0.94)	< 0.0001
UNK	0.99 (0.98–1.01)	0.255

*Note:* Estimates reflect the mean ratios of exponentiated beta coefficients for each term in the log linear model in comparison to the reference group, where the mean ratio is the factor change in BW associated with each race or ancestry group, compared to the referent group. For example, a mean ratio of 0.99 reflects a 1% decrease in BW associated with American Indian/Alaskan Native status, compared to White infants.

Abbreviations: AFR, African; AMR, American; EAS, East Asian; SAS, South Asian; UNK, unknown.

## 4 Discussion

### 4.1 Main Findings

In a cohort of well‐characterised nulliparous pregnant people, a GRS_BW_ derived from a set of previously published BW‐associated SNPs was modestly associated with infant BW. However, its association with BW was not statistically significant among participants who self‐identified as Black, Asian, or had an unknown race, or among those with AFR, EAS, SAS, or UNK genetically predicted ancestry. We also found that the magnitude of association between GRS_BW_ and BW varied widely across groups. Our findings suggest that the GRS_BW_ does not fully generalise to racially or genetically diverse groups.

### 4.2 Interpretation

Our findings are concordant with other studies assessing the relationship between race and foetal growth. Across a variety of contexts, studies have found that race is associated with differences in foetal growth among both unselected and low risk groups [19, 20, 21]. In our study, both self‐identified race and genetically predicted ancestry were associated with gestational age‐adjusted BW, even after controlling for sex and GRS_BW_. Our finding that the GRS_BW_ was not consistently associated with BW across non‐European ancestry groups is also consistent with existing studies of other conditions. Polygenic risk scores derived in primarily European cohorts perform significantly less well in participants of non‐European descent for multiple conditions, including venous thromboembolism, coronary artery disease, heart disease, hypertension, chronic kidney disease, and cancer [22, 23, 24, 25, 26, 27, 28]. The non‐generalizability of genetic findings to diverse populations is a critical gap with the potential to exacerbate existing disparities [29, 30]. Our findings add to this important body of work by extending it to foetal growth, which holds considerable clinical relevance in perinatal medicine.

The significance of differing magnitudes of association is difficult to interpret, since the association was not significant in most groups, and we did not find direct evidence of effect modification of the GRS_BW_ ‐BW association by race or predicted ancestry. While an assessment of the clinical utility of this GRS_BW_ is beyond the scope of this study, it is worth noting that the overall magnitude of the association of the GRS_BW_ with BW was relatively small. The mean ratio estimates represent the factor change in the BW for a change in the independent variable, where an increase in GRS_BW_ of one unit (1.0) is associated with a 7% increase in BW. However, the entire range of computed GRS values in the cohort (−0.214 to 0.713) is less than 1.0, making the typical GRS_BW_‐associated difference much smaller than 7%, which may not be clinically significant.

Our findings have several implications for future efforts in this area. First, our results demonstrating that GRS_BW_ is not associated with BW in many ancestry groups, and that genetically predicted ancestry remains independently associated with BW after controlling for GRS_BW_, suggest that additional work is needed to achieve equity in the performance of genetic risk scores for BW prediction. Methods to support multi‐ancestry polygenic risk score derivation are now available and are promising in their ability to equitably leverage genotypes for trait prediction. However, such methods still depend on the availability of discovery cohorts that themselves are diverse, if not globally representative [31, 32, 33, 34, 35]. As precision medicine advances, it may improve recognition of diseases such as foetal growth restriction and thereby allow for earlier surveillance or treatment. However, creating and validating genetic risk scores in EUR‐only geographic ancestral cohorts, as has largely been done to date, ensures that the resultant scores do not include the full breadth of human genetic variation. This decreases the utility of the score for all individuals and could create or exacerbate inequities in adverse pregnancy outcomes because any inaccuracies would disproportionately accumulate among those from excluded genetic ancestry groups. Furthermore, while race has no inherent genetic meaning, exclusion of the ancestral populations (e.g., AFR) that significantly contribute to genetic variation within some racial groups increases the risk that such incomplete scores perform disproportionately more poorly. For instance, in our study, the predicted genetic ancestry for individuals identifying as Black/African American was 98% AFR. The two are not synonymous and cannot be interchanged. Simply stated, this overlap demonstrates the genetic clustering within AFR ancestry of certain phenotypic features (notably skin colour), which also contributes to the US social construct of Blackness. However, as genetic variation is greater within than between racial groups, exclusion of this genetic ancestry pool perpetuates structural and systematic reasons that GRS scores may perform more poorly among some Black individuals. Indeed, a GRS that reflects the breadth of genetic variation across all ancestral groups would be likely to perform better for individuals from all race categories, as it would include the broadest identification of genetic contributors to foetal growth.

Second, our results suggest that there may be additional unaccounted‐for factors linking race to foetal growth, as self‐identified race remains associated with BW after controlling for GRS_BW_. This is overall unsurprising, as racial categories themselves have no inherent genetic meaning. As a social construct, however, the persistent association between race and BW could represent the influence of systematic differences in environmental and social exposures that are known to contribute to racial health disparities. It is also plausible that epigenetic differences may encode the intergenerational impacts of racism and other forms of oppression and hardship imposed on minoritized populations. However, the GRS_BW_ used in our study, like most genetic risk scores, cannot meaningfully capture epigenetic changes. These factors and their complex relationships to the genetics of foetal growth warrant further investigation. Our study also reinforces the importance of clearly disentangling genetics from the sociostructural construct of race when investigating health outcomes.

### 4.3 Strengths and Limitations

Strengths of our study included the use of a large, multicentre U.S. obstetric cohort with geographical and racial diversity. The nuMoM2b protocol provides both standardised specimen collection and validated outcomes ascertainment, and we used externally derived BW‐associated SNPs for GRS_BW_ assessment, adding to the rigour and validity of our analysis. Additionally, our assessment of GRS_BW_ using two distinct approaches (both self‐identified race and genetically predicted continental ancestry groups) demonstrates that the lack of generalizability is a robust finding.

Our study also had limitations. The need to map SNPs derived from reference build GRCh37 to GRCh38, ultimately leading to the use of 72 rather than 86 SNPs may have reduced the strength of the overall association between the GRS_BW_ and BW. Also, it is possible that the lack of association between the GRS_BW_ and BW is due to the sample sizes of each group, especially for the smallest groups, such as Native Hawaiian/Pacific Islander or American Indian/Alaska Native, which each had just 32 and 17 participants, respectively. However, sample size limitations are unlikely to fully explain the lack of association, as the GRS_BW_ was associated with BW in the multiracial group (*n* = 508) and was very nearly significant among those of SAS predicted ancestry (*n* = 274), both of which had smaller sample sizes than the largest groups in which GRS_BW_ was not associated with BW. Finally, the investigators who identified the BW‐associated SNPs and their weights did not compile them into a GRS to use as we have, such that we cannot directly compare our results with theirs. However, our study nonetheless quantifies the degree to which genetic findings identified in EUR‐only cohorts do not fully generalise to groups of other backgrounds.

## 5 Conclusion

A GRS_BW_ derived from a European‐ancestry cohort was not consistently associated with BW across self‐identified race or genetically predicted ancestry groups, suggesting a lack of generalizability to populations of non‐White race and non‐European ancestry. These findings highlight the need for both globally representative genetic discovery cohorts and the use of analytical approaches that can incorporate the social and structural factors that drive racial inequities in foetal growth. Such changes could maximise generalizability of genetic risk methods for disease prediction and improve equity in access and treatment. Prioritising the generalizability of genetic findings is critical to prevent exacerbation of longstanding health inequities by creating and perpetuating personalised medicine approaches that have inequitable utility.

## Author Contributions

Each author fulfills the requirements for authorship, with contributions as follows: parent cohort study design and execution: R.M.S., D.H., G.S.; study conception, design, and planning: N.R.B., L.P., M.J., J.M.; data analysis: L.P., M.J., B.M.; results interpretation: B.T.‐F., L.P., M.Z., M.P.D., R.G., B.M., R.M.S., D.H., T.W., J.G.S., G.S., N.R.B.; manuscript drafting: B.T.‐F., N.B.; manuscript editing and final approval: B.T.‐F., L.P., M.J., M.Z., M.P.D., J.M., R.G., B.M., R.M.S., T.W., D.H., J.G.S., G.S., N.B.

## Funding

This work was supported by NICHD (1 K08 HD113835‐01, U10 HD063020, U10 HD063037, U10 HD063041, U10 HD063046, U10 HD063047, U10 HD063048, U10 HD063053, U10 HD063072, R01HD101246), University of Utah One Utah Data Science Hub. The computational resources used were partially funded by the NIH Shared Instrumentation Grant 1S10OD021644‐01A1. DNA extraction and genotyping were funded by Indiana University Grand Challenges Precision Health Initiative.

## Ethics Statement

This work was designated by the University of Utah Institutional Review Board (protocol # 00113635) as exempt based on the U.S. Department of Health and Human Services 45 CFR 46 definition of human subjects' research.

## Conflicts of Interest

The authors declare no conflicts of interest.

## Supporting information


**Table S1:** bjo70088‐sup‐0001‐TableS1.xlsx.

**Table S2:** bjo70088‐sup‐0002‐TableS2.xlsx.

**Table S3:** bjo70088‐sup‐0003‐TableS3.docx.

## Data Availability

The data that support the findings of this study are available from the corresponding author upon reasonable request.
