# Supplementary material for: Generalisability of Maternal Genetic Risk Score for Birth Weight Across Racial Identity and Ancestry: A Secondary Analysis of a Prospective Cohort Study
Source: BJOG. 2025 Nov 30;133(4):708–15. doi: 10.1111/1471-0528.70088 (PMC12884248; doi:10.1111/1471-0528.70088)
Supplement: Supplementary file 3 — Table S3: bjo70088‐sup‐0003‐TableS3.docx. [file BJO-133-708-s002.docx]

**Supplementary material.**

**Supp. Table 3.** Association between infant birth weight and maternal GRS_BW_, infant sex, and gestational age at delivery, nuMoM2b cohort, 2010-2013

| **Parameter** | **Estimate (95% CI)** | **t value** | **p** |
| --- | --- | --- | --- |
| **Intercept** | -1.164 (-1.561, -0.767) | -5.74 | <.0001 |
| **Maternal GRS_BW_** | 0.063 (0.041, 0.085) | 5.69 | <.0001 |
| **Female sex (vs male)** | -0.042 (-0.047, -0.036) | -14.34 | <.0001 |
| **Gestational age** | 0.419 (0.397, 0.441) | 37.49 | <.0001 |
| **Gestational age^2^** | -0.005 (-0.005, -0.004) | -30.14 | <.0001 |

CI= Confidence Interval

GRS**_BW_** = Growth restriction score for birth weight
